# Ligand scaffold hopping combining 3D maximal substructure search and molecular similarity

**DOI:** 10.1186/1471-2105-10-245

**Published:** 2009-08-11

**Authors:** Flavien Quintus, Olivier Sperandio, Julien Grynberg, Michel Petitjean, Pierre Tuffery

**Affiliations:** 1 MTi, RPBS, INSERM UMR-S973, Université Paris Diderot-Paris 7, F-75013, Paris, France; 2 MTi, CDithem, INSERM UMR-S973, Université Paris Diderot-Paris 7, F-75013, Paris, France; 3 DSV/iBiTec-S/SB2SM, CNRS URA 2096, CEA Saclay, F-91191 Gif-sur-Yvette, France

## Abstract

**Background:**

Virtual screening methods are now well established as effective to identify hit and lead candidates and are fully integrated in most drug discovery programs. Ligand-based approaches make use of physico-chemical, structural and energetics properties of known active compounds to search large chemical libraries for related and novel chemotypes. While 2D-similarity search tools are known to be fast and efficient, the use of 3D-similarity search methods can be very valuable to many research projects as integration of "3D knowledge" can facilitate the identification of not only related molecules but also of chemicals possessing distant scaffolds as compared to the query and therefore be more inclined to scaffolds hopping. To date, very few methods performing this task are easily available to the scientific community.

**Results:**

We introduce a new approach (LigCSRre) to the 3D ligand similarity search of drug candidates. It combines a 3D maximum common substructure search algorithm independent on atom order with a tunable description of atomic compatibilities to prune the search and increase its physico-chemical relevance. We show, on 47 experimentally validated active compounds across five protein targets having different specificities, that for single compound search, the approach is able to recover on average 52% of the co-actives in the top 1% of the ranked list which is better than gold standards of the field. Moreover, the combination of several runs on a single protein target using different query active compounds shows a remarkable improvement in enrichment. Such Results demonstrate LigCSRre as a valuable tool for ligand-based screening.

**Conclusion:**

LigCSRre constitutes a new efficient and generic approach to the 3D similarity screening of small compounds, whose flexible design opens the door to many enhancements. The program is freely available to the academics for non-profit research at: .

## Background

Drug discovery remains a lengthy and costly process in which *in silico *approaches have been proven of interest to help to reduce the cycle time and cost, as well as to increase the productivity [1], in complement to experimental techniques such as high-throughput screening (HTS) [2], high-throughput X-ray crystallography [3], or combinatorial chemistry [4]. There are two main computer-based approaches.

Structure-based virtual screening (SBVS) approaches depend on the knowledge of the 3D structure of the target. They aim at docking collections of small compounds in the target structure, resulting in a quantified interaction score to identify candidate compounds [5]. Ligand-based virtual screening (LBVS) approaches [6] are based on the assumption that structurally similar compounds are likely to exhibit similar biological activities. They are often used when at least one compound biological activity is proven, but detailed structural information on the mechanisms underlying the biological activity is not available. This might come since the biological target is totally unknown, or since no structural information about the drug-target interaction could be obtained. When a significant number of structure activity relationship data have been validated, one can apply QSAR (Quantitative Structure Activity Relationship) techniques [7]. The goal of such techniques is to derive from the available 2D or 3D data a statistical model that can be used to predict new active molecules. Other LBVS methods focus on similarity searches, and encompass 2D-similarity-search (e.g. [8]), shape-based (or 3D-based) (e.g. [9-12]) and pharmacophore based techniques [13]. The latter approach relies on the knowledge of the biological activity of multiple hits to identify key features for the search. It has been extensively explored, and its relevance has been assessed by many studies. 2D- and 3D-based approaches attempt to quantify compound similarity based on the sharing of chemical groups, the 3D shape of compounds and their chemistry. Most of the similarity search approaches developed so far are available as commercial packages. ChemMine [8] is a pure 2D-similarity searching tool which uses a classic Tanimoto coefficient as a scoring criterion. MED-SuMoLig [10] and ROCS/ROCS-cff [11] are 3D-similarity search approaches. While relying on different underlying concepts, they combine both shape and chemistry to mine chemical compounds. Both of these 3D approaches need a multiconformational representation of the chemical library to be screened because their algorithm treat the small molecules as rigid entities. Some other methods such as Surflex-sim [14] can treat the molecules with flexibility but are not suited for large in silico screening due to computational time limitations.

Concerning LBVS approaches, one important outcome from previous studies is the importance of the balance between search specificity and search diversity. Search specificity can be related to receptor selectivity, which would result in restraining the search close to the bioactive compounds, whereas diversity is related to the necessary alleviation to any scaffolds dependency, to propose new relevant scaffolds divergent if possible from the known bioactive compounds. Here, we introduce a new approach (LigCSRre) to mine chemical libraries based on molecular similarity with a query potent compound. It explicitly addresses the two former points of view. It is based on a genuine 3D maximum common substructure – 3D similarity – search engine CSR [15] that is capable of identifying a three-dimensional match between two sets of atoms, the query set, and those of a chemical library. The nature and type of the atoms is taken into account through a set of rules using Unix regular expression formalism that makes possible to tune the nature of the atoms allowed to be eligible for pairing in the CSR engine, thus enhancing the physico-chemical relevance of the 3D similarity search. Those rules are user-defined, which makes this program totally customizable. Whereas similar formalism has already been introduced, although using a different search engine, for protein similarity search [16] using PDB atom and residue naming conventions, we extend here its usage to the combination of atomic types of sybyl mol2 , i.e. to more detailed physico chemical typing of atoms.

In order to validate our approach, we have applied LigCSRre on a previously reported test set that contain several bioactive ligand queries (through 6 different protein targets) among about 38 000 drug-like molecules used as decoy molecules. Here, we used 5 targets and 47 active compounds, described in [10] (10 on CDK2, 9 on FX, 10 on NA, 8 on RNase, and 10 on TK), an approach similar to that of Sheridan and co-workers [17] which also deals with the variation between multiple active compounds. Also, in order to explore the performance of the approach, it seemed important to use co-crystallized ligands since the availability of the 3D information allows to investigate in detail the relevance of the superposition. Indeed, to evaluate the performance of our program we assessed two general aspects of 3D-ligand-based screening tools, superimposability and enrichment. The former characterizes the ability of such tool to correctly align co-active ligands of a same protein target, and the latter assesses whether or not a higher score is given to co-active molecules versus decoy molecules. Early enrichment, that is, the ability for a virtual screening tool to present in the very top ranked molecules the most potent compounds is particularly important when the experimental screening capacity is only few hundred molecules. Because we use in the present study the same test set that the one used in [10], we can apply a reference protocol for both superimposability and enrichment, in order to compare our program to the three other commercial packages used, MED-SuMoLig [10], ROCS/ROCS-cff [11], and ChemMine [8]. Finally, we also analyse its performance when combining the mining of collections using several compounds independently, i.e. in an opposite direction to the pharmacophore approach.

## Results and discussion

### 3D superimposability

As a first assessment of our program capabilities in screening, we decided to test its ability to correctly align (or superimpose) co-active molecules with respect to each other. It is very important that 3D-ligand-based screening methods be accurate in such alignment because a series of structural conclusions can be driven from them. The co-localization of key chemical groups can help to design pharmacophores and in the end facilitate the hit identification or even the optimization of lead compounds. Therefore, we ran our program for all the actives (here 47 compounds) using their respective bioactive X ray conformation as the query compound. Our first control was evidently to assess whether LigCSRre was capable of retrieving the closest de novo conformation of the query molecule itself, which was systematically the case. Then we sought for the closest de novo conformation of the N-1 other co-active molecules on the same protein target. We defined as experimental alignment or experimental superimposition the experimentally derived superimposition of the co-crystallized ligands by superimposing the protein structures from which they were extracted. Table 1 – upper results – presents the results per family. On average, the superpositions of the other co-actives onto the active query were coherent with the experimental alignment 71% of the time (RMSD < 2 Å between the chosen superimposed de novo conformation and the bioactive conformation plus visual inspection to ensure that the ligands are properly aligned – not flipped over), which indicates that LigCSRre recovered true positives (co-actives) for the right reason and not thanks to a misalignment. For the other cases, we observed some alignment flipped over with regard to the query due to molecular symmetry. We also observed mis-alignments resulting from targeting one or several peripheral groups rather than the global architecture of the query ligand.

**Table 1:** LigCSRre 3D superimposability and enrichment performance

	CDK2	FXa	NA	RNAse	TK	All
3D sup.	0.61	0.58	0.73	0.76	0.73	0.71

Enrichment						
1%	0.28	0.20	0.68	1.0	0.43	0.52
3%	0.40	0.27	0.80	1.0	0.55	0.60
5%	0.44	0.30	0.82	1.0	0.63	0.64
10%	0.49	0.42	0.89	1.0	0.73	0.71

For 5 families of active compounds, we present the LigCSRre performance reached for 3D superimposability and for enrichment at various thresholds.

**Figure 1:**
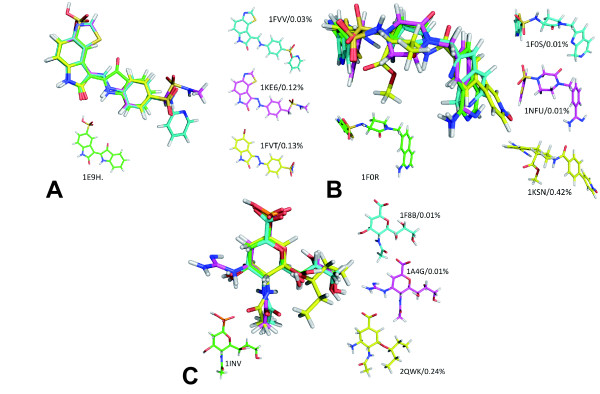
**Best co-active hit alignments**. The green carbon molecules represent the query molecule, cyan-, pink- and yellow-carbon molecules are respectively the 1st, 2nd and 3rd co-active molecules found by LigCSRre. Panel A (CDK2), panel B (FXa), panel C (NA).

It must be noted that the pursuit of maximum superimposition has a meaning only on the parts of the ligand that both interact an identical region of the protein. Thus, two parts of the ligands to be superimposed that would point toward the solvent, outside of the binding pocket and that would not be correctly superimposed, would certainly not represent a failure from the superimposing tool. Figure 1 illustrates LigCSRre behaviour for 3 series of targets, CDK2, FXa, and RNase. The query molecules (green carbon compound) are simply the first molecule on each subset, that is, not necessarily the best active (by best active we mean the one that could catch the highest number of co-actives in the smallest database percentage level of subsetting). For CDK2 the query molecule was extracted from structure 1E9H and is based on an Indirubin scaffolds (two indole system rings), which is the only biindole-based ligand of the CDK2 subset. One clearly sees that the superpositions goes beyond the sole indole function they have in common which is an interesting proof of scaffolds hopping. Indeed, the superimposed parts of the hits on the query correspond to the segment of the molecules that interact with the well-known "hinge" region of the CDK2 protein, essential for tight binding to CDK2 through a network of up to 3 Hydrogen bonds (left bottom corner of the CDK2 panel). The high chemical diversity of the FXa is a primary challenge to most of the ligand-based methods (see paragraph on comparison to tiers program). The query molecule on Figure 1 was extracted from PDB structure 1F0R. LigCSRre managed to identify the global structural feature of the compounds, that is, a haliphatic ring surrounded by two ring-based arms and used it to superimpose the 3 FXA hits onto 1F0R. 1F0S ligand was correctly aligned onto the query, but unfortunately, it flipped over the 2 next hits, 1NFU and 1KSN. 1F0R and 1F0S ligands shared most their chemical structures except for some minor variation for the portion of the ligands that go deep inside the P1 pocket of FXa, such that our ligand-based tool had no problem to detect the 1F0S ligand as a one of the first hits and to correctly align it onto the query 1F0R ligand. On the contrary, 1KSN and 1NFU ligands do not share chemical features with 1F0R ligand, different groups interacting with the P1 pocket, different linker, and different group interacting with the entrance of the binding site (exposed to the solvent). So, any ligand-based tool cannot rely on a strong chemical similarity or even topological similarity to proceed to an accurate similarity detection between the query 1F0R ligand and 1KSN, 1NFU ligands. Concerning NA, the query ligand was extracted from structure 1INV. It is the only NA ligand of the subset without a carboxylic function attached to the core 6-atom ring. Despite the lack of carboxylic function, LigCSRre managed to correctly orient the different hits onto the NA ligand query. To summarize, we found that LigCSRre is able to globally perform structural alignment in a satisfactory fashion by selecting in vast majority (71% of the time) the closest de novo conformation of the query co-active compounds.

### Enrichment Assessments – Independent query runs

**Figure 2:**
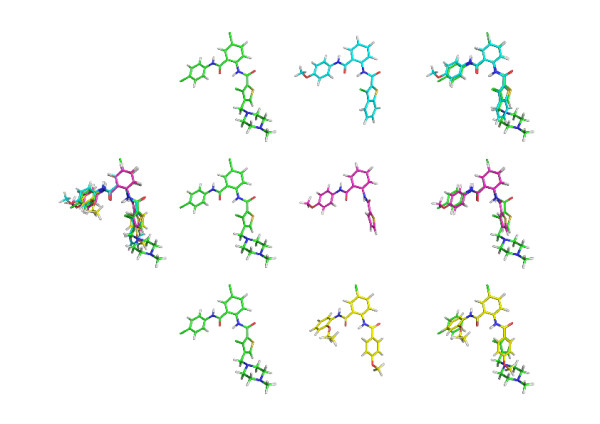
**FXa **(1MQ5) **first ranked decoys**. Superimposition of some of the very first ranked decoy molecules on one FXa active (1MQ5).

To be consistent with a previous study [10], we evaluated the enrichment capacities of LigCSRre using an identical protocol. Average results are reported Table 1, enrichment section. As seen from Table 1, the 1% enrichment value is of 0.52, which indicates that more than one half the active compounds are scored within the first 1% compounds. This is important since the essence of virtual screening tools is to avoid an experimental testing of the full length database but rather to select an early enrichment subset of compounds enriched in putative actives. Early enrichment is therefore of primary importance when dealing with large chemical database because the experimental and financial capacity of certain research departments is just sufficient to assess a few hundred molecules. However, we also note large variations depending on compound families. RNase ligands gave by far the best results with 100% of recovery before 0.1% level of subsetting for each active of the subset. For FXa, the corresponding value is of only 20%. Looking more in detail at FXa results, we observed large variations among the different active compounds. For instance, LigCSRre managed to recover about 40% of the co-actives for 3 of the active queries within 1% level of subsetting. The FXa subset represented a real challenge for 6 ligands which display poor recovering rates. The first explanation is again the chemical diversity and the lack of consensual physico-chemical properties with respect to the binding interactions with the protein. In the present case the program could not systematically discriminate actives from decoys. To illustrate the difficulty to discriminate between FXa actives and decoys we have studied the best ranked decoys for the FXa LigCSRre runs. As seen on Figure 2 one can see that the very first decoy molecules identified by LigCSRre for the 1MQ5 run represent chemical structures that could be easily found similar with the naked eye of a medicinal chemist. The presence on the query ligand of ortho-substituted phenyl ring hanging two amide functions followed by another phenyl and a thiophen is correctly identify in three decoy molecules and logically superimposed onto the query. Those decoy molecules present more chemical similarity to the query ligand than some of the other co-actives of the FXa subset. In this example, the part of the ligand which interacts with the well known P1 pocket of Factor Xa is not the basic piperazine but rather the bromo-phenyl on the other side. While basic groups are preferentially expected to bind the P1 pocket it is surely not a systematic rule, in this case this hydrophobic group can be accommodated as well. So here, we look preferentially for molecular similarity with the bromo-phenyl part of the ligand and with the linker, which is exactly what shows the results of this run, with even a supplementary molecular similarity with the linker of the query ligand for the 3 hits shown in the figure. Interestingly, the present example gives the opportunity to search for a rarer type of FXa inhibitors as opposed to the regular benzamidine or guanidinium group that are usually expected to interact with the bottom of the P1 pocket.

Another difficult case was the CDK2 subset that is rather difficult because of its chemical diversity. LigCSRre managed however to undertake some scaffolds hopping (e.g between 1E9H and 1FVV ligands), and other examples of scaffolds hopping could be cited like between 1OGU and 1H1S ligands. The 3 first CDK2 hits, for the 1E9H run, were obtained in a percentage level of subsetting inferior to 0.15% of the total test set (i.e within the top 60 of the ranked test set). This means that, using an initial bank of 38 000 molecules, the experimental testing of the top 60 molecules would have provided already 3 new active compounds, and not necessarily with the same scaffolds. For the other CDK2 ligands, all actives recovered at least 2 and 3 co-actives at 1 and 5% of subsetting respectively, which represent a moderate performance in term of global enrichment but still offers interesting tracks for hit identification at early enrichment. The NA and TK ligands gave convincing results. The results for NA show good recovering rates with 3 actives (1A4G, 2QWK and 1F8B ligands) that managed to identify 90% of the co-actives at 0.1% of subsetting, and 2 more actives (adding 1IVB and 1INF ligands) at 3% of subsetting getting 90% of the actives as well. For TK, 7/10 TK ligands managed to recover more than a third of the co-actives in the top 1% of the ranked database. Interestingly, the three ligands that only manage to do so at higher percentages level of subsetting are those having a guanidine-like structure (2KI5, 1KI2 and 1KI3) rather than a thymidine-based scaffolds, which represents another case of scaffolds hopping. So, our method is capable of identifying molecules with a relatively distant scaffolds but at the same time of having the discriminatory power to distinguish a molecule belonging to a different subclass.

To summarize, one can state that LigCSRre has a rather flexible behavior with respect the ligand chemical diversity and flexibility. As illustrated for RNase, NA, and TK ligands, both the superimposition and enrichment results display the robust behavior of a rather 2D-search based method that is capable of very good performances on chemical series having a shared chemical core, regardless of the strong presence of decoy compounds. On the other hand, results for CDK2, some of the RNase and TK ligands, and to some extent for certain ligands of FXa, LigCSRre show enough plasticity in both the superimposition and the enrichments to offer scaffolds hoping capabilities with respect to more diversified chemistries.

Finally, it is interesting to examine the variation of each approach depending on the individual ligands. Table 2 reports for each target, minimal and maximal values for enrichment scores at 1 and 3%. One clearly sees that the variations can be very large. This is observed for all methods, on the same order of variation. For FXa, it is noticeable that, for all methods, scores can be as low as 0% for some ligand and as high than 75% for MED-SuMoLig. Even for well established method such as ROCS-cff, one sees that at 3% enrichment, we observe an enrichment variation from 29 to 86% for RNAse, depending on the query. These observations highlight the importance to assess the scaffolds hopping capabilities of 3D ligand-based methods using multiple 3D queries rather than a single one.

**Table 2:** Compared enrichment variations over 5 methods

	CDK2	FXa	NA	RNAse	TK
Enrichment 1%					
CSR	11–56	0–63	33–100	100-100	22–78
SM	22–67	0–63	44–67	43–100	22–78
RC	11–56	0–38	67–100	29–57	44–100
2D	0–33	0–38	22–89	100-100	22–89

Enrichment 3%					
CSR	33–56	0–63	56–100	100-100	33–89
SM	22–67	0–75	56–100	43–100	56–100
RC	22–67	0–63	78–100	29–86	56–100
2D	11–56	0–38	22–89	100-100	44–100

For 5 families of active compounds, we present for LigCSRre (CSR), MED-SuMoLig (SM), ROCS-cff (RC) and ChemMine (2D), the minimal and maximal enrichment scores at 1% and 3%. Results expressed in % of recovered co-active ligands.

### Enrichment Assessments – Combined query runs

**Figure 3:**
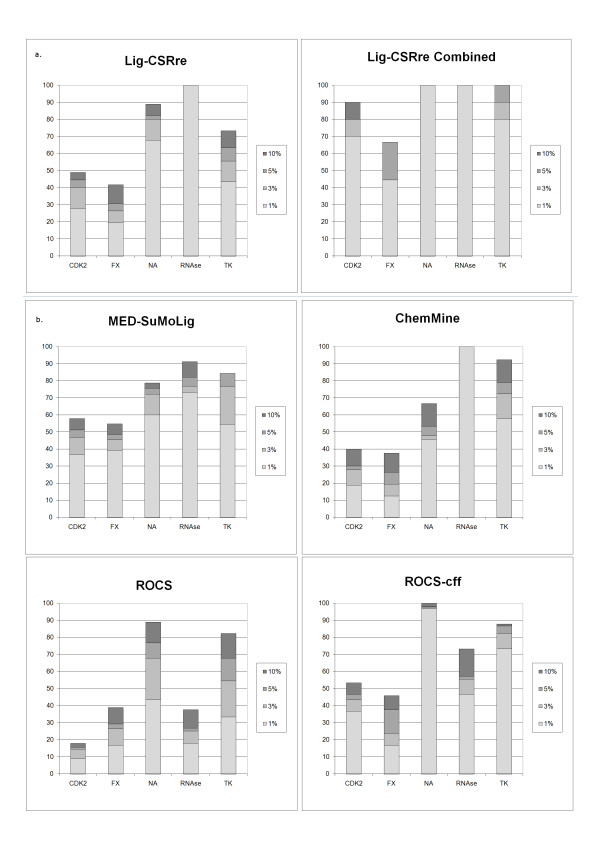
**Comparison with related methods**. Comparison of the percentage of recovered co-active compounds at 1, 3, 5, and 10% subsetting with three software assessed on the same dataset.

One interesting observation that could be made about individual enrichment curves is that the enrichment is highly dependent on the ligand query. This is particularly true for Neuraminidase, for which the results at 0.1% of subsetting are either at 100% or 23% of recovered active depending on ligand query, 1A4G or 1INV ligands respectively. The fact that full ligand structures are used as LigCSRre query rather than pharmacophoric hypothesis makes the program more dependent on the chemical properties of each query, and might inevitably bring noise to the results. Nonetheless, this approach has the advantage to avoid focusing on what co-actives must have in common for activity (pharmacophore) but rather on what might bring specificity to protein binding. Thus, by combining several query searches a higher number of hits and therefore a higher number of therapeutic tracks will be opened. This is the essence of the group fusion techniques used in 2D search approaches [18]. It is clear on Figure 3 (panel a – combined) that shows the results of cumulating the different queries of a same protein subset by taking the best score for each molecule (actives + decoys) across the N query runs (e.g CDK2; N = 10). In that case the enrichment rate we obtained is much higher. We think this shows a convincing example of the benefit of cumulating the available information of several actives to retrieve complementary novel chemical entities rather than focusing on consensual chemical features.

### Sensitivity to compound conformational sampling

As discussed in previous studies related to SVBS [19,20], or to LVBS [21], 3D search techniques can be dependent on the number of conformations per compound. In particular, McGaughey et al. [21] have shown that ROCScff performances are not deeply affected by increasing the number of conformations per compound up to 100. Because the enrichment rates were more modest for CDK2 and FXa, we decided to measure the impact of the number of conformers by generating a new database this time with a maximum number of 100 conformers. This way we would identify how tightly the enrichment rate is correlated to the relative flexibility of the compounds. In the case of FXa the number of conformers for the actives has a consequence on the enrichment rates, but only at 10% subsetting, therefore showing a rather low impact on early enrichment. The FXa ligands are quite flexible 5 to 10 rotatable bonds, it is therefore not surprising that an increased number of conformers be more efficient, although the search must face an increasing noise from decoy structure flexibility. For CDK2 the impact on enrichment is rather low. This can be explained by the lower flexibility of the CDK2 ligand versus those of FXa, which obviously makes the dependence to the number of conformers milder for CDK2 ligands. In that case the availability of more CDK2 conformers is balanced by the increasing presence of decoy conformers in terms of enrichments. These results tend to show that while ligand flexibility is of primary importance for larger ligand at higher percentage level of subsetting, it does not seem to influence the behavior of our program at early percentages of enrichments. It is therefore interesting to see that for the very first ranked hits of LigCSRre a maximum of 50 conformers is sufficient to obtain what would obtained with higher maximum number of conformers.

### Comparison with related methods

Because we use the same test set as used in [10] we were able to directly compare the averaged enrichment performance of our product with the three ligand-based packages, MED-SuMoLig, ROCS/ROCS-cff, and ChemMine that were used in that study. We have focused our study on 5 of the 6 proteins they used because the ligands of HIV-1 protease do not display drug-like properties. Indeed, the ligands have a molecular weight and number of rotatable bonds significantly above some of the Lipinski and Veber's standards: MW < 500 and Nrotatable < 10, respectively [Lipinski et al; Veber et al.].

The cumulated percentage of actives recovered are displayed on Figure 3 at four thresholds of percentage level of subsetting, 1%, 3%, 5% and 10%. For example, for CDK2 on average 3 actives out of 9 (10 ligands CDK2 – 1) are recovered in the top 1% of the ranked bank, that is here 1% of 38 000 compounds, therefore before rank 380th. On average across the 5 targets, the results show that LigCSRre recovered 52% of the co-actives in the top 1% of the ranked list, whereas MED-SuMoLig, ChemMine, ROCS-cff and ROCS display respectively 51%, 42%, 50% and 22%. Hence, LigCSRre performs, on average slightly better than the other methods for early enrichment.

When comparing the enrichment rate for CDK2 ligands we could see that LigCSRre is more powerful than a simple 2D-search similarity such as ChemMine at all level of subsetting, with on average 10 more percents in favor of LigCSRre. The performances are similar to the program ROCS-cff program at higher level of subsetting 3, 5, and 10 and a little under at 1%, while MED-SuMoLig is the one that performs the best on this protein target. It is not surprising that an approach like MED-SuMoLig essentially based on pharmacophoric properties (Hydrogen bond donor, Hydrogen bond acceptor, etc) performs well on such ligands when one considers the very importance of the ligand interaction with the "hinge region" of the protein through a network of up to 3 Hydrogen bonds.

Concerning FXa, the results of all 4 methods are modest due to the chemical complexity of those ligands and the absence of consensual features. Both LigCSRre and ChemMine have more problem than MED-SuMoLig and ROCS-cff to recover the active at all percentage of subsetting. But interestingly, at 1% level of subsetting, i.e at early level of enrichment, LigCSRre and ROCS-cff perform similarly. So this means that even in the case of a complicated target such as FXa, very early enrichment can be observed, not necessarily with the majority of the co-actives but some of them.

LigCSRre performed very well for NA with about 70% of the active recovered at 1% level of subsetting (i.e rank 380th), and up to about 90% at 10% of the ranked databank. ROCS-cff clearly outperforms the other programs on this target with nearly 100% enrichment at 1% level of subsetting, while MED-SuMoLig's performance are slightly lower than those of LigCSRre, ChemMine performing the poorest with 45% enrichment at 1% level of subsetting, which is satisfactory at this level.

For the TK ligands, all methods perform similarly at 10% subsetting, around 80% of recovered co-actives, except for ChemMine that reaches 90%. The results of ChemMine can be explained by the high chemical similarity of the TK ligands, their small size and their low flexibility. At early enrichment level (1% subsetting), LigCSRre is a little behind the other methods but with a satisfactory 45% of recovered co-actives, while MED-SuMoLig, ROCS-cff and ChemMine display a recovering percentage of co-actives of, 55, 72, 58%, respectively. Even though the results of LigCSRre are still quite satisfactory, one can note the relative superiority of ROCS-cff at early level of enrichment. Indeed, it is the only method tested that penalizes molecular discrepancies besides identifying molecular similarities. Discrepancies become more pronounced when the query molecule is of small size, increasing the probability for the three other methods to find a bigger hit molecule containing a substructure compatible with the query structure. This is illustrated by the poor results for ROCS that does not take into account the molecular chemistry in addition of the shape. TK was one of the only two cases with NA where ROCS alone had satisfactory results, but both targets have relatively small ligands, and both ROCS versions penalize molecular discrepancies.

For the RNase ligands, both LigCSRre and ChemMine reach the perfect recovering percentage possible (100%) at only 1% level of subsetting. We can see that despite the high similarity of the RNase ligands MED-SuMoLig does not have an equivalent enrichment on this protein target, even if at 70% it remains quite satisfactory. ROCS-cff has the poorest performances on RNase with an average of 45% of recovered co-actives. One explanation of the present discrepancy between the programs is linked to their global concept and to the structures of the RNase ligands. Two of the RNase ligands display a bicephal structure (1QHC and 1JN4 ligands), one part based on the classic purine-like scaffolds, and the other based on a pyrimidine-like scaffolds, the two parts being connected by a poly-phosphate chain linker. From a chemical point of view, one faces a chemical consistency across the RNase actives because of the purine-based part the two ligands have in common with the rest of the RNase ligands. But from both a topological (ROCS concept) and a pharmaco-topological (MED-SuMoLig) point of view, this represents a more important difference because the two bi-cephal ligands are twice as big as the rest of the ligands. Moreover, one of the RNase ligand has a different mode of binding while being purine-based (1O0O ligand) as well, such that the 3D distribution of the query atoms is quite different. This represents a challenge especially for methods such as ROCS, because of the shape-associated properties of the query ligand versus the co-actives. Even if LigCSRre is also a 3D method in essence, it also has, as MED-SuMoLig, the discriminatory power to identify rigid chemical entities such as a purine-based scaffolds that are also well described by more simple 2D patterns (like in ChemMine). Finally one of the ligand is exclusively based on a pyrimidine structure (1O0N) with ribose and phosphate associated. In the X ray crystallographic structures the pyrimidine part of this ligand superimposes perfectly with the pyrimidine part of the bicephal ligands cited above. This means, that to be accurate a similarity search using 1O0N-ligand as the query would retrieve only the bicephal ligands (that also possesses a pyrimidine-based structure) and not the only purine-based ligands as done by LigCSRre and ChemMine which obtained 100% of recovered co-actives. This raises the complicated question of recovering true positive ligands but with the wrong alignment. In the case of RNase a very minor chemical change in the ligand can trigger a flipping over of the purine scaffolds with respect to the classic binding mode while the ligands still have a very high degree of similarity. This problem can be associated in the case of RNase-like proteins to the so-called reverse binding mode issue [22-25] for which several binding modes can be observed for one ligand.

Finally, it is interesting to examine the variation of each approach depending on the individual ligands. Table 2 reports for each target, minimal and maximal values for enrichment scores at 1 and 3%. One clearly sees that the variations can be very large. This is observed for all methods, on the same order of variation. For FXa, it is noticeable that, for all methods, scores can be as low as 0% for some ligand and as high than 75% for MED-SuMoLig. Even for well established method such as ROCS-cff, one sees that at 3% enrichment, we observe an enrichment variation from 29 to 86% for RNAse, depending on the query. These observations highlight the importance to assess the scaffolds hopping capabilities of 3D ligand-based methods using multiple 3D queries rather than a single one.

### Future perspectives

In order to improve the discrimatory power of our program towards false positives, we have decided to address the problematic of penalizing molecular discrepancies besides identifying molecular similarities as programs such as ROCS do. We have tried to penalize hit molecules that had too many heavy atoms with respect to the query molecule. This has increased the results for small ligands such as TK ligands for which a 10% improvement was observed at 10% level of subsetting. The observation could not be made for all protein targets though. So a more sophisticated criterion could be applied in order to improve the enrichment rates. The regular expressions used to determine the rules of atom pairings offer enough plasticity to construct more complex rules such that the final match between query and hit molecules could be more discriminate towards false positives.

## Conclusion

We have introduced a new free flexible approach for small compounds 3D molecular similarity screening that explicitely addresses both aspects of 3D and physico-chemical similarity. Compared to gold standard of the field, it proves able to achieve efficient early enrichment in active compounds. Due to its flexible design, many perspectives now range from scanning generic collections to deriving focused collections specific rules.

## Methods

### Compound test sets

As a reference bank, we have considered the filtered version of the chembridge diversity set (50 000 compounds) , a set already used in previous studies [10]. The dataset has been filtered for ADME/tox properties [26]. The remaining diversity set of Chembridge contains 37 907 molecules different molecules with the following properties: a molecular weight ranging between 200 and 900, a computed octanol/water partition coefficient (logP) between -5.0 and 6.0, a polar surface area between 0.0 and 160, a maximum number of rotatable bonds of 20, a number of Hydrogen bond donor ranging between 0 and 8, a number of Hydrogen bond acceptors ranging between 0 and 12, and at least two heteroatoms per compound. To limit the deviation from standard Lipinski rule of five, we only tolerate one Lipinski violation. Each compound has a multiconformer representation, setting up a maximum of 50 conformers for the dvs50 set and 100 conformers to the dvs100 set. The dvs-50 set contains about 1 150 000 molecules, which makes an average of 30 conformers per molecule. For query active compounds, we have used series of experimentally validated inhibitors, RiboNuclease (RNAse) (8 ligands), coagulation factor ten (FXa – 9 ligands) and the Cyclin Dependent Kinase 2 (CDK2 – 10 ligands), Neuraminidase (NA – 10 ligands), and Thymidine Kinase (TK – 10 ligands) for which the experimental structure of the complex exists, which makes a total of 47 active compounds that have a multiconformational representation as well. These active compounds display similar physico-chemical properties as the compounds of the remaining dataset [27]. CDK2 and FXa ligands are rather chemically diverse with respect to the NA, RNase, and TK ligands. TK ligands are quite small and represent thymidine analogs for the most part and guanidine-based for the rest of them, NA ligands are built around a consistent 6-atom ring with various peripheral groups, whereas RNase ligands are based on analogs to purine-based or pyrimidine-based compounds or both (for two of the RNase actives) and are rather flexible molecules (7–10 rotatable bonds).

### Similarity search

The similarity search engine, LigCSRre is an evolution of the CSR algorithm originally developped by M. Petitjean [15]. The CSR algorithm searches for the maximal 3D motif common – or maximal substructure (MSS) – to two sets of coordinates. Whereas other approaches such as SQ [28] or CLIP [29] use clique detection approaches, the CSR algorithm is a parameter free approach that iteratively and stochastically searches for the largest set of atom pairings between two clouds of atom coordinates – no a priori pairings or a priori rules such as the knowledge of the neighbors are required. Atomic natures, bonds and connectivity information are ignored. Briefly, each iteration starts from a random initial superposition, and iterative pairings of atoms are performed until no new pairing occurs. Pairing is based on distance sort of the N1*N2 interatomic distances between the N1 atoms of the molecule 1 and the N2 atoms of the molecule 2. The array of the N1*N2 distances is sorted by increasing values. The first atom-pair, corresponding to the smallest distances, is always included in the common motif. Next pairs are included until a member of a pair already included in the common motif occurs. The latter pair is not included in the motif and the pairing terminates. Then the complete sets of coordinates are best superimposed from the current pairings and the whole process distance sorting/best fit is iterated until no new pair is accepted. This whole process is performed for a series of random starting points and CSR returns the largest motif identified. CSR was shown efficient to retrieve similarities in large sets of coordinates. However, limitations occur since similarities between biological molecules must also consider that atomic properties of the pairs are compatible.

In LigCSRre, we have implemented several additional particular features. Firstly, LigCSRre accepts, similarly to Escan [16], a regular expression formalism that allows, for each atom, to define which pairings are possible based on some physicochemical properties (see next section). This results in smaller search space and increases search efficiency – LigCSRre usually requires much less iterations than CSR. Secondly, LigCSRre extends the set of pairings at search convergence. The extended pair collection embeds the MSS identified by the CSR algorithm enlarged by atom pairs that are distant by less than a user specified tolerance. This can result in more relevant similarity search since it is possible that CSR stops to enlarge its MSS for an atom already paired, but hiding subsequent pairs.

Since atom nature deeply condition the chemical properties of compounds, and therefore their binding to receptors, it is important to have some control on the atomic types that can be paired during the similarity search. The authorized or forbidden pairings must be defined in a way flexible enough to be adapted to a particular chemical context, or to express the knowledge of an expert for some particular family of compounds. To take into account these considerations, we use the Sybyl mol2 atom types, as assigned by open-babel [30], and we use a three level mechanism of regular expressions to define atomic types compatible for pairing. The first one is the default level: the atoms are assigned a default regular expression that will authorize them to be paired only with atom having the same atomic type. The second level is the generic level that defines equivalence classes. At this level, it is for instance possible to assert that a carbon atom could be paired with any carbon, but not oxygen or sulfur. The third level is the specific level. This level makes possible to attach a regular expression to a particular atom of a particular compound. For instance, it would make possible to define, for a specific carbon of known importance for chemical activity, to accept that it could match only an aromatic carbon or a Nitrogen. The precedence order gives the higher priority to the specific level, then to the generic level, then to the default level. In this study, since we want to assess the generic performance of the approach, we have not used the specific level. Instead, we have defined a minimal set of rules for the generic level. More in detail the equivalence classes correspond (i) to carbons but carbo-cations, (ii) sp2 Oxygen (0.2 and 0.co2 mol2 types) (iii) sulfoxide and sulfone Sulfur (S.o and S.o2); (iv) sp2 and sp3 Sulfur, and (v) Nitrogen. For other atomic types, the default rules only accept pairings for atoms of identical atomic types. The LigCSRre algorithm is iteratively applied to each compound of the bank. For each, we store the number of bonds (nB) identified as shared, where a bond is denoted as shared if both two atoms at bond extremities are paired on exit of LigCSRre. We also store the size of the pairing set (nP), and the RMS deviation associated with these pairs (RMSd). Once all the compounds have been screened, they are sorted using a cascading procedure: according to nB, then to RMSd.

### Multipe screening merging

Given a collection of compounds sharing the same inhibitor activity, we merge the results using a best rank criterion: The lists obtained for each compound independently are read from the first position to their last, and each bank compound is inserted in the merge resulting list on the basis of its first occurrence.

### Enrichment measurement

To measure the compound enrichment, we use as criterion *Ef *= *nAf*/(*nA*-1), where *nA *is the number of active compounds of the inhibitor family, and *nAf *is the number of active compounds (but the query) retrieved in the best of percent of the sorted results.

## Authors' contributions

FQ has implemented the regular expression scheme specific of the small compounds. JG and PT have rewritten in C the original CSR algorithm developed by MP, and interfaced it with the regular expression facilities. OS has designed the test sets and the approach benchmark. PT has initiated and supervised this study.
